# Mitochondrial DNA Reveals the Trace of the Ancient Settlers of a Violently Devastated Late Bronze and Iron Ages Village

**DOI:** 10.1371/journal.pone.0155342

**Published:** 2016-05-13

**Authors:** Carolina Núñez, Miriam Baeta, Sergio Cardoso, Leire Palencia-Madrid, Noemí García-Romero, Armando Llanos, Marian M. de Pancorbo

**Affiliations:** 1 BIOMICs Research Group, Centro de Investigación “Lascaray” Ikergunea, Universidad del País Vasco (UPV/EHU), Vitoria-Gasteiz, Spain; 2 Instituto Alavés de Arqueología, Vitoria-Gasteiz, Spain; Universitat Pompeu Fabra, SPAIN

## Abstract

La Hoya (Alava, Basque Country) was one of the most important villages of the Late Bronze and Iron Ages of the north of the Iberian Peninsula, until it was violently devastated around the 4th century and abandoned in the 3rd century B.C. Archaeological evidences suggest that descendants from La Hoya placed their new settlement in a nearby hill, which gave rise to the current village of Laguardia. In this study, we have traced the genetic imprints of the extinct inhabitants of La Hoya through the analysis of maternal lineages. In particular, we have analyzed the mitochondrial DNA (mtDNA) control region of 41 human remains recovered from the archaeological site for comparison with a sample of 51 individuals from the geographically close present-day population of Laguardia, as well as 56 individuals of the general population of the province of Alava, where the archaeological site and Laguardia village are located. MtDNA haplotypes were successfully obtained in 25 out of 41 ancient samples, and 14 different haplotypes were identified. The major mtDNA subhaplogroups observed in La Hoya were H1, H3, J1 and U5, which show a distinctive frequency pattern in the autochthonous populations of the north of the Iberian Peninsula. Approximate Bayesian Computation analysis was performed to test the most likely model for the local demographic history. The results did not sustain a genealogical continuity between Laguardia and La Hoya at the haplotype level, although factors such as sampling effects, recent admixture events, and genetic bottlenecks need to be considered. Likewise, the highly similar subhaplogroup composition detected between La Hoya and Laguardia and Alava populations do not allow us to reject a maternal genetic continuity in the human groups of the area since at least the Iron Age to present times. Broader analyses, based on a larger collection of samples and genetic markers, would be required to study fine-scale population events in these human groups.

## Introduction

**Fig 1:**
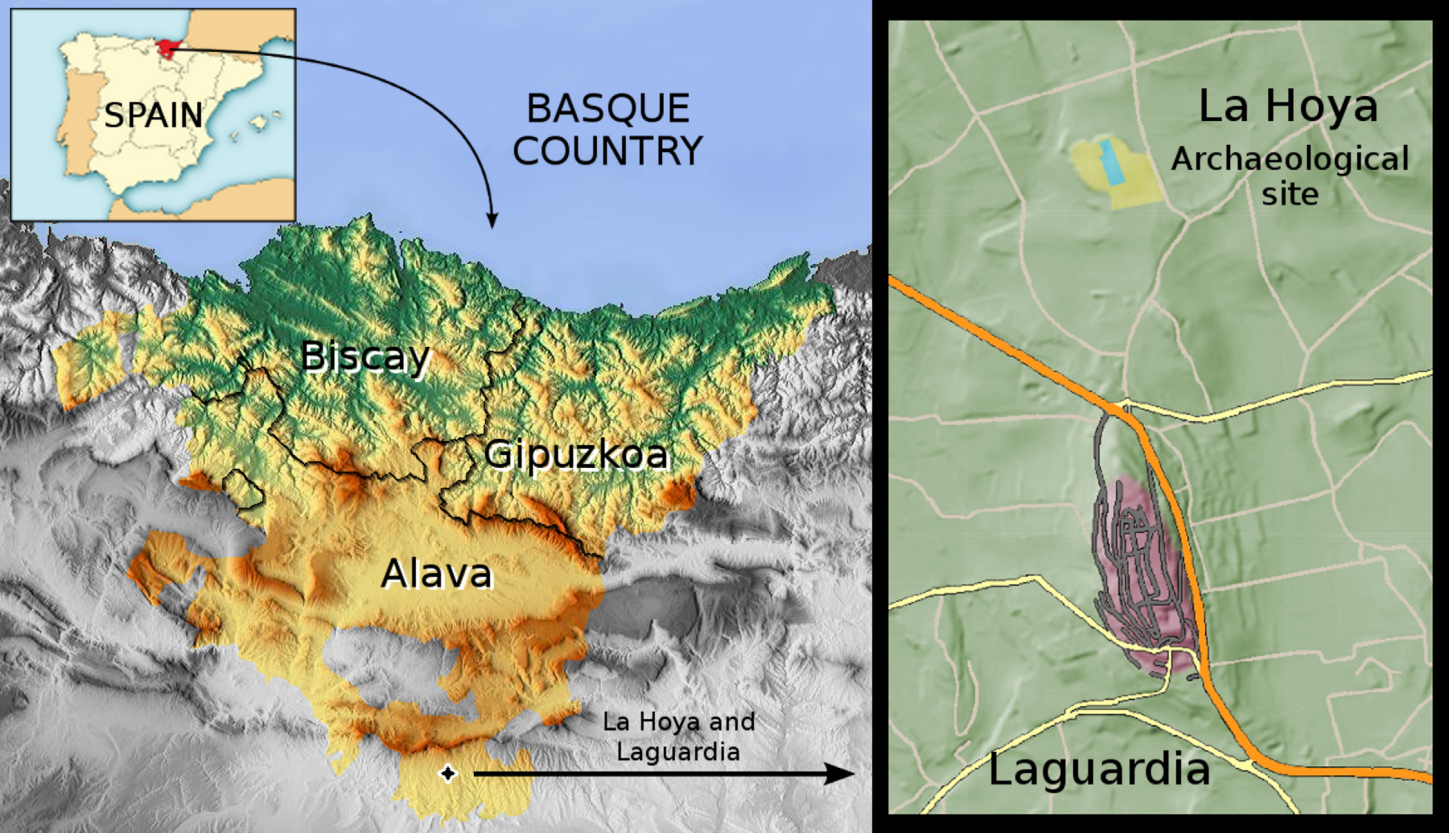
Enlarged view of the Basque Country and its provinces with the location of the village of Laguardia and La Hoya archaeological site (distance 1 km).

La Hoya was a settlement located in the region currently known as the Basque Country, in Northern Spain, from Late Bronze to Iron Ages (15th to 3rd centuries B.C). This fortified town located near Laguardia (Alava) (Fig 1) had an extension of approximately four hectares and was inhabited during 13 centuries, becoming an important Celtiberian trade center during the Iron Age. The first human settlement dated back to the 15th century B.C., when Indo-European populations from Central Europe made contact with the local megalithic cultures [1]. This interaction has been revealed by several evidences from the Indo-European culture found in the lower levels of the archaeological excavation [2]. Later on, the village experienced a period of intense change involving the introduction of Celtiberian influences from the Iberian plateau, which promoted important social, economical, and technological advances. Around the 4th century B.C., La Hoya was violently destroyed by enemies who burned a considerable area of the village and killed part of its population. Despite its posterior rebuilding, La Hoya never recovered its previous splendor and the site was permanently abandoned in the 3rd century B.C. due to unknown reasons [1]. Archaeological evidences suggest that the inhabitants of La Hoya settled in a nearby hill, where nowadays the village of Laguardia is located (Fig 1).

In the present study we analyzed the mtDNA hypervariable regions HVS-I and HVS-II of 41 human remains recovered in La Hoya archaeological site, as well as the complete mtDNA control region of the present-day populations of the village of Laguardia and the province of Alava. Laguardia is the extant population settled nearby the ancient village of La Hoya and Alava is a representative sample set of the province. The comparison between the ancient population of La Hoya and these extant Basque populations might allow assessing the maternal genetic continuity of these populations settled in the same area but separated by thousands of years.

## Materials and Methods

### Archaeological site of La Hoya

The archaeological excavation carried out during 1973 and 1989 revealed more than 260 human remains from different cultural periods [1]. The highest number of interments was recovered in the more recent Celtiberian levels with a total of 131 individuals, while in the previous Indo-European levels 49 individuals were found. The remaining 80 remains were not attributed to any specific level.

### Archaeological samples

**Fig 2:**
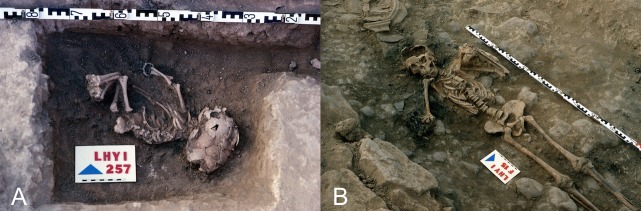
**Human skeletal remains of an infant (A) and an adult individual (B) found in La Hoya archaeological site**.

A total of 41 human remains from the Celtiberian Iron Age (5th century B.C.), belonging to 33 newborns or infants (30 femora, 2 humeri, and 1 skull fragment) (S1–S3 Figs) and 8 adults (5 teeth, 2 femora, and 1 skull fragment) (Fig 2), were selected for genetic analyses from the archaeological collection of La Hoya, deposited in *BIBAT Museum*, the Archaeological Museum of Vitoria-Gasteiz (Alava, Basque Country).

All necessary permits were obtained for the described study, which complied with all relevant regulations. The genetic analysis of these ancient remains was granted by the institutions that are in charge of the collection (*BIBAT Museum* and *Instituto Alavés de Arqueología*) and approved by the Director of Cultural Heritage of the Department of Culture of the Basque Government, as issued by the resolution of 2nd August 2011.

### Modern Samples

Mouthwash samples were collected from 107 autochthonous Basque individuals from Alava province in the Basque Country (Northern Spain): 51 from Laguardia (LG) and 56 from the whole Alava province (AL). Voluntary donors were healthy and maternally unrelated individuals, whose ancestors (until the third generation back) were born in Alava or, specifically, in Laguardia for the 51 LG samples. All participants gave their written informed consent to participate in this study following the ethical standards of the Helsinki declaration. This study was approved with the favorable ethical report from the Faculty of Pharmacy of the University of the Basque Country, signed at 26th September 2008.

### Contamination Precautions

To prevent and detect contamination, we followed precautions at all stages of the analysis [3]. Appropriate laboratory clothing (disposable laboratory coveralls, masks, caps, glasses, shoe covers, and gloves) was used by all the analysts. All procedures were carried out under maximum sterile conditions in a specialized facility with separate laboratories for specimen handling, pre-polymerase chain reaction (PCR), and post-PCR analyses. DNA-free certified reagents were used, and materials and solutions were properly decontaminated (UV exposure/autoclaving). Workbenches and laboratory equipment were frequently treated with 50% bleach. Laboratory contamination was monitored with extraction blanks and negative control reactions. Repetitions of extractions and amplifications were performed in order to corroborate the results. Only samples without any contamination in all stages were considered for further analyses.

### DNA extraction and quantification

To eliminate possible contaminants from prior handling, the outer layer of the bones was removed with a sanding machine and teeth were cleaned with 10% bleach. Subsequently, bone fragments and teeth were 30 min UV-irradiated each side. Samples were then grounded into a fine powder in a 6750 Freezer Mill (Spex SamplePrep, USA) under liquid nitrogen to avoid DNA degradation. Afterwards, about 500 mg of powder was used for a silica-based DNA extraction method (Qiagen, Hilden, Germany) (modified of [4]) adapted to the Hi-Flow^®^ DNA Purification Spin Columns (Generon, Berkshire, UK). A final concentration step to 30–40 μl was carried out using Amicon-30 columns (Millipore, MA, USA). Extracted DNA was quantified by real-time quantitative PCR (qPCR) using Quantifiler^TM^ Human DNA Quantification kit (AB/LT/TFS: Applied Biosystems^TM^, Life Technologies, ThermoFisher Scientific, Waltham, MA, USA) in an ABI PRISM 7000 Sequence Detection System (AB/LT/TFS) following the manufacturer’s recommendations.

DNA extraction from modern samples was performed in a separate laboratory room, using the standard phenol: chloroform procedure [5]. DNA was quantified with the Quant-iT PicoGreen dsDNA Assay Kit (Invitrogen, Carlsbad, USA) in a DTX880 Multimode Detector (Beckman Coulter, Fullerton, USA).

### Mitochondrial DNA analysis

Hypervariable segments I (HVS-I) and II (HVS-II) of the mtDNA control region were analyzed in all 41 samples from La Hoya. Additionally, hypervariable segment III (HVS-III) was also analyzed in some samples in order to deepen into haplogroup J subclassification. PCRs contained 2.5 μl Buffer 10X, 0.75 μl MgCl_2_ (50 mM), 0.5 μl dNTPs mix (2.5 mM), 1 μl BSA (10 mg/ml), 0.15 μl Taq (5 U/ μL) (Bioline, UK), 0.25 μl of each primer (10 μM), 3 μl of DNA sample, and sterile water up to a total volume of 25 μl. Amplification of each hypervariable segment was carried out using three overlapping pairs of primers, except for HVS-III for which one primer pair was used (S1 Table). Cycling parameters were 95°C for 3 minutes, followed by 37 cycles of 95°C for 45 seconds, 55°C for 60 seconds, and 72°C for 60 seconds, and a final extension of 72°C for 10 minutes.

Amplification products were subjected to electrophoresis on 1.5% agarose gels and visualized with GelRed (3% μL/ml) (Biotium Inc., Hayward, USA) and UV light (UVItec Cambridge). DNA products were purified using 0.5 μl EXO (Exonuclease I) and 2.5 μl SAP (Shrimp Alkaline Phosphatase) (Takara Bio Inc., Japan) to 10 μl of PCR product and sequenced in both directions using the same PCR primers. Sequencing reactions were performed with BigDye^®^ Terminator^TM^ v3.1 Cycle Sequencing Kit (AB/LT/TFS). Sequencing products were purified with BigDye^®^ XTerminator^TM^ Purification Kit (AB/LT/TFS).

Capillary electrophoresis was performed on a 3130 Genetic Analyzer (AB/LT/TFS). Sequencing reaction products were analyzed with the Sequencing Analysis software (AB/LT/TFS). Sequences were aligned to the revised Cambridge Reference Sequence (rCRS) [6] using SeqScape^®^ Software v2.5 (AB/LT/TFS). Edition ranges are shown in S4 Table. Haplogroups were assigned following the mtDNA phylogeny (www.phylotree.org, v16) [7].

Complete mtDNA control region (CR) from modern samples was amplified, sequenced, and interpreted as reported in [8]. All samples containing length heteroplasmies were assigned based on the majority molecule. All these sequences have been deposited into EMPOP under the accession number EMP00556. Likewise, sequences are available online at GenBank under accession numbers JX669021—JX669071 (Laguardia) and JX669072—JX669127 (Alava province).

### Haplogroup minisequencing

Ancient and modern samples assigned to R0 macrohaplogroup were additionally analyzed by minisequencing for subtyping the following H lineages: H1 (G3010A), H1bh (C11377T), H2a (C4769T), H3 (A6776G), H4 (C3992T), H5’36 (G456A), H6a (C3915T), H7 (T4793C), H8 (A13101C), H9 (T13020C), H10 (T14470A), H11 (A8448G), H12 (C3936T), H13a1 (A4745G), and H15 (T6253C). Amplification and minisequencing primers used are described in [9], except for H1 and H3 amplification primers that are defined in [10]. Those R0 samples not classified into any of the abovementioned H subhaplogroups were additionally analyzed for 7028 and 11467 diagnostic coding region positions for H and U, respectively (S1 Table).

### Data analysis

In order to statistically evaluate differences in DNA yield and inhibition among adult and infantile samples we performed two chi-square tests based on type of sample (adult/child) and absence/presence of DNA or inhibition, using the PAST v3.06 software [11]. In the case of DNA yield, we classified into the category of “presence of DNA” those samples with a DNA yield above the detection limit of Quantifiler^TM^ Human Quantification kit (≤0.023 ng/μl).

To carry out comparative analyses, population data available in the literature of ancient populations from the Iberian Peninsula were compiled (S2 Table). A principal component analysis (PCA) based on mtDNA haplogroup frequencies was carried out among La Hoya and ancient Iberian populations. For this purpose, archaeological sites with at least 9 individuals were chosen and subhaplogroups were grouped in their basal haplogroup since not all archaeological samples had the same haplogroup resolution. PCA analysis was conducted with PAST v3.06 software.

Diversity parameters for the complete mtDNA CR of Laguardia and Alava populations were calculated using the Arlequin Software v3.5 [12]. We also used this software to examine population differentiation from the mtDNA point of view by computing F_ST_ values based on haplotypes. Additional Basque populations from Alava, Gipuzkoa, Bizkaia, and Navarre were included in these calculations for comparison purposes to a local level (S2 Table). The length polymorphisms of the poly-C stretches at HVS-I, HVS-II, and HVS-III were disregarded in the analyses.

Approximate Bayesian Computation (ABC), implemented in DIYABC 2.1 [14] was used to test the most likely model for the local demographic history that best fits the data. Two competing scenarios were designed including the three populations herein studied (S4 Fig). Scenario 1 consists of a hypothesis assuming that genealogical continuity between the ancient population of La Hoya and the modern population of Laguardia existed, whereas Alava is not derived from this ancient population. Scenario 2 assumed that the ancient and modern populations are genealogically independent.

Uniform distributions for the historical parameters were defined. The mutation model chosen was the Hasegawa–Kishino–Yano model [16], as recommended by [17], as the most appropriate model to describe the sequence evolution of the mtDNA control region, assuming a per-site and generation mutation rate ranging uniformly between 1 x 10^−6^ and 1 x 10^−8^ mutations. Only the simulations generating summary statistics close to the observed were considered to estimate the posterior probabilities of the models and parameters. We performed the summary statistics for populations pairs based on F_ST_ [15]. A total of 3 x 10^6^ simulated data sets were used. Pre-evaluation of each scenario was performed by a PCA, based on the summary statistics, and checking the congruence between the observed and simulated data set. Logistic regression was used to compare posterior probabilities. Performance of the scenario parameters was assessed by means of type I (false positives) and type II (false negative) errors, and by computing the relative bias. We estimated the type I error probability as the proportion of cases where the selected scenario did not show the highest posterior probability compared with the competing scenario for the simulated datasets generated under the best-supported model [18]. Likewise, the type II error probability was calculated for the alternative scenario, and determining the proportion of cases where the best-supported model was incorrectly chosen as the most probable model.

## Results

### Quantitative PCR analysis

DNA from eight adults and 33 newborn and infant remains was extracted and quantified. Real-time qPCR analysis provided valuable information about DNA preservation, as well as possible presence of inhibitors in the samples. Positive quantification results were obtained in 15 out of 41 samples, with nuclear DNA yields up to 0.2010 ng/μl (S3 Table). Two out of eight adult samples and four out of 33 child samples yielded an amount of DNA above the quantification detection limit. Additionally, inhibition was detected in a total of 25 samples (threshold cycle or Ct >30 or undetermined). More specifically, inhibition was only detected in one out of eight adult samples, and in 24 out of 33 of child samples.

A chi-square test was performed in order to evaluate the differences observed in adult and child samples for DNA recovery and inhibition. No significant differences between adult and child samples based on the recovery of DNA (p = 0.355) were observed, but the same test based on absence or presence of inhibition did present significant differences (p<0.002). This fact could be related to the type of bone available and consequently analyzed. Infantile samples comprised small bones that easily disintegrated, and whose thin and fragile cortical layer hindered the sample processing.

Due to the limited nuclear DNA recovery efficiency (36.59%) a strategy based on the analysis of short amplicons (164–207 bp) of mtDNA was followed for the 41 samples included in the study.

### MtDNA haplotypes of La Hoya ancient human remains

MtDNA haplotypes were obtained in 25 of the 41 studied samples (60.98% of the total), with different range of sequence coverage achieved (S4 Table). Neither amplifiable DNA nor reproducible results were obtained for the rest of samples. A total of 14 different haplotypes were identified (based on common range), seven of which were observed more than once. These results lead us to assume the presence of some potential maternally related individuals. However, the number of shared haplotypes might be overestimated due to the limited size of the common range.

Samples LHY099 and LHY181 shared the same HVS-I and HVS-II haplotype (16069T, 16126C, 73G, 185A, 188G, 228A, 263G, 295T, 309.1C, 315.1C), indicating a possible common maternal ancestry. In addition, the aforementioned HVS-II haplotype was also observed in samples LHY112, LHY158, and LHY170. Furthermore, other 13 samples shared common (partial) haplotypes: LHY249/LHY223/LHY227, LHY235/LHY236, LHY104/LHY245, LHY163/LHY213, LHY169/LHY244, and LHY097/LHY258. In some of these cases, the limited sequence range accomplished for HVS-I and HVS-II does not allow concluding a maternal relationship among them. Interestingly, two of these individuals, LHY235 and LHY236, were found in the same burial group, further supporting a maternal kinship between these samples, which correspond to two infants. The rest of individuals sharing mtDNA haplotypes were found in different burial places, nevertheless this not necessarily excludes a common maternal ancestry.

### MtDNA haplogroups of La Hoya ancient human remains

The major haplogroups found in La Hoya were H and J, which were present in nine individuals each, being the subhaplogroups H1 (six individuals) and J1c2 (five individuals) the most prevalent ones (Table 1). Haplogroup U5 was also noticeably represented in the sample set (five individuals), particularly sublineages U5a1b1 and U5b1c1a (two individuals each). Haplogroups K2a5 and W were each identified in a single individual.

**Table 1:** Haplogroups identified in the sample set of La Hoya. N = number of individuals.

Haplogroup	N
H1	6
H3	3
J1*	1
J1c1	2
J1c2	5
K2a5	1
U5a1b1	2
U5b*	1
U5b1c1a	2
W	1
Total	24

As previously mentioned, samples LHY235 and LHY236 sharing the same haplotype and belonging to J1 haplogroup were found in the same burial group. Thus, they were considered only once (S4 Table) due to a possible maternal kinship relationship.

The high presence of haplogroup J in the ancient remains of La Hoya is striking even though previous studies have already pointed out the high frequency of J and, probably, J1 lineages in the Basque Country [19], since the Neolithic [20].

### Comparison of La Hoya lineages with other ancient populations from the Iberian Peninsula

**Fig 3:**
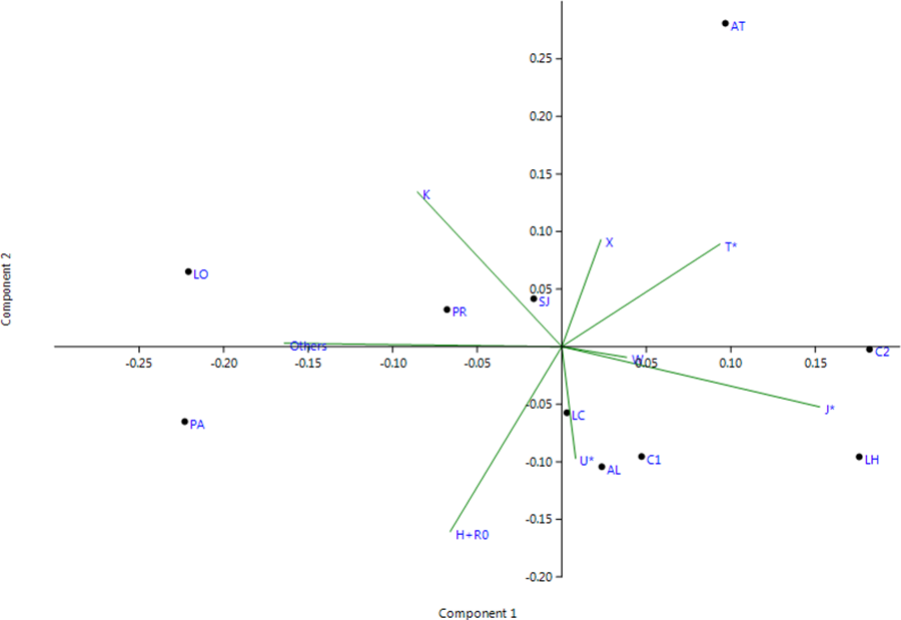
Principal Component Analysis (PCA) based on haplogroup frequencies of La Hoya archaeological samples (LH) and other historic and prehistoric populations from the literature. Neolithic: Los Cascajos, Navarre (LC); Paternanbidea, Navarre (PA); Camí de Can Grau, Barcelona (C1); San Juan Ante Portam Latinam, Basque Country (SJ). Chalcolitchic: Pico Ramos, Basque Country (PR); Longar, Navarre (LO); El Mirador Cave, Atapuerca (AT). Pre-Roman: Girona, Catalonia (C2). Post-Roman: Aldaieta, Basque Country (AL).

In order to evaluate the haplogroup variability of La Hoya mtDNA lineages in a geographically and temporarily context, haplogroup frequencies were compared with those of other ancient historic and prehistoric populations from the Iberian Peninsula (S2 Table). Fig 3 shows the first two components of the multivariate (PC) analysis performed (69.16% of the overall variability) on these ancient Iberian populations.

The position of La Hoya (LH) in the PCA was mostly determined by the comparatively high frequency of haplogroup J and haplogroup R0 within the ancient context. The cemetery of Aldaieta (AL) also located in the Alava region and chronologically more recent (VI-VII A.D.) with regard to La Hoya, showed similar frequencies of haplogroups J and H. The same occurred with the pre-Roman and Neolithic archaeological sites of Catalonia (C1 and C2), although C2 is also influenced by the frequencies of haplogroups T and W. Moreover, the Basque Neolithic Los Cascajos (LC) shows a similar pattern too. The Neolithic and Chalcolithic sites of the Basque Country and Navarre, Pico Ramos (PR), Paternanbidea (PA), Longar (LO), and San Juan Ante Portam Latinam (SJ), along the component 2, harbour similar frequencies of haplogroups R0 and K. Finally, the sample from the Mirador Cave (AT) in Atapuerca shows marked differences in haplogroup frequencies with respect to the rest of ancient sites, especially in terms of haplogroups T and X.

### MtDNA haplotypes of present-day Alavese populations

Complete mtDNA CR was obtained from two present-day populations from the village of Laguardia and the whole province of Alava (S5 Table). A total of 39 different haplotypes were observed in the 51 individuals from Laguardia sample set, being 29 haplotypes unique. In the population of Alava, 43 haplotypes were identified in the 56 individuals studied, and seven of them were shared among different individuals.

Diversity parameters for Laguardia and Alava are shown in Table 2. The diversity observed in both populations (*H* = 0.9882 ± 0.0064 and *H* = 0.9851 ± 0.0077, respectively) is in accordance with the genetic variability described in other autochthonous populations from Basque Country (*H* = 0.9795 ± 0.0054 in [8]; see also S6 Table). However, these values remain far from diversity levels registered for other Europeans [19]. In fact, the Basque population has been pointed out as one of the group with less genetic diversity in Europe. This low genetic diversity is directly related to the isolated nature of the Basque people, since endogamy, genetic drift, bottlenecks, and other evolutionary processes may have a great impact in its genetic variability.

**Table 2:** Summary of diversity parameters for 51 mtDNA CR (16024–576) sequences from Laguardia and 56 from Alava region. Insertions at 16193, 309, and 573 were ignored for all calculations.

Population statistics	Laguardia (N = 51)	Alava (N = 56)
Different haplotypes	39	43
Shared haplotypes	10	7
Genetic diversity (*H*)	0.9882 ± 0.0064	0.9851 ± 0.0077
Nucleotide diveristy (π_n_)	0.0073 ± 0.0038	0.0055 ± 0.0030
Mean pairwise differences (π)	8.2235 ± 3.8758	6.2487 ± 3.0115

Population comparison based on F_ST_ analysis between Laguardia, Alava and other Basque Country populations from previous results from our group [8] was performed to evaluate the genetic similarity at the local level (S6 Table). In order to avoid sampling bias, the Alava population sample from the present study and Alava from [8] were grouped as they did not showed significant differences. F_ST_ analysis showed a low (F_ST_ = 0.0099) and non significant (p>0.005, after Bonferroni correction for multiple comparisons) genetic distance for Laguardia and the Alava population sample. On the other hand, these two population groups showed significant differences (p<0.005) with the rest of Basque populations. However, the F_ST_ values as well as the limited sample size are not high enough to evidence a strong population substructure.

### MtDNA haplogroups of present-day populations

**Fig 4:**
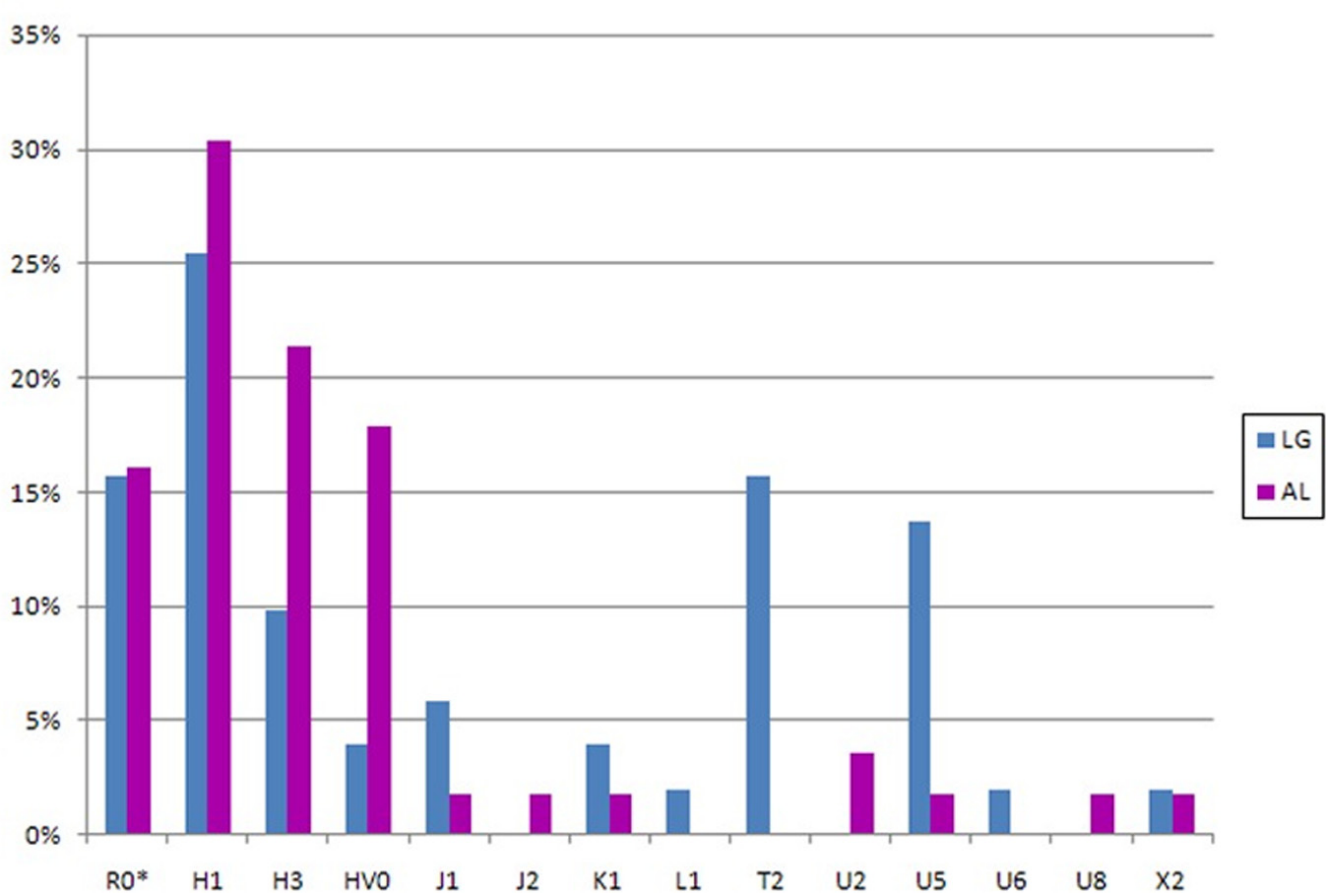
MtDNA haplogroup composition of Laguardia (LG) and Alava (AL) population sample sets. R0* includes H and HV lineages except for H1, H3, and HV0.

The classification of lineages into haplogroups revealed that native populations of Laguardia and Alava are solely represented by European lineages (Fig 4), except for two individuals belonging to L1b and U6a lineages found in Laguardia. The most frequent haplogroup in these populations was R0, as it is observed in most Western-European populations. The frequency of R0 (excluding HV0 clade) in Laguardia (51%) is in agreement with previous results on Basques [19]. Interestingly, the value obtained in Alava sample set (67.9%) represents the highest frequency of this clade reported to date and it is far from the average frequency of West Eurasia (40%) [20]. Likewise, it is noticeable the presence of two of the highly frequent lineages in the Basque region such as subhaplogroup H1, which presents a frequency of 25.5% in Laguardia and 30.4% in Alava; and H3 represented by 9.8% in Laguardia and 21.4% in Alava. Other subhaplogroups, which have also been described in the contemporary Basque population, such as U5, J1 or J2, present a variable percentage in these two populations (Fig 4).

Laguardia and Alava populations share lineages belonging to HV0, J1, K1, U5, and X2 haplogroups, as well as from macro-haplogroup R0. Additionally, it is noticeably the identification in Laguardia of two individuals belonging to L1b and U6a lineages, which are found in African populations [22, 23]. The presence of African lineages in the Iberian Peninsula could derive from the historic Islamic occupation [24, 25] or have prehistoric roots [26]. Other possible scenario is that these lineages would have entered to the Peninsula along with return migrants from the American continent.

### Population affinities between La Hoya and two present-day populations of Alava

The comparative analysis between La Hoya and the current populations of Laguardia and Alava did not show complete haplotype coincidences, except for lineages belonging to haplogroup H. Nonetheless, phylogenetically close haplotypes were identified, for instance the lineage J1c2 of LHY099/LHY181 (16069T, 16126C, 73G, 185A, 188G, 228A, 263G, 295T), LG003 (16069T, 16126C, 16278T, 16366T, 73G, 185A, 188G, 228A, 263G, 295T), and VG110 (16069T, 16126C, 16278T, 16291Y, 16366T, 73G, 185A, 188G, 228A, 263G, 295T) individuals from La Hoya, Laguardia, and Alava, respectively.

**Fig 5:**
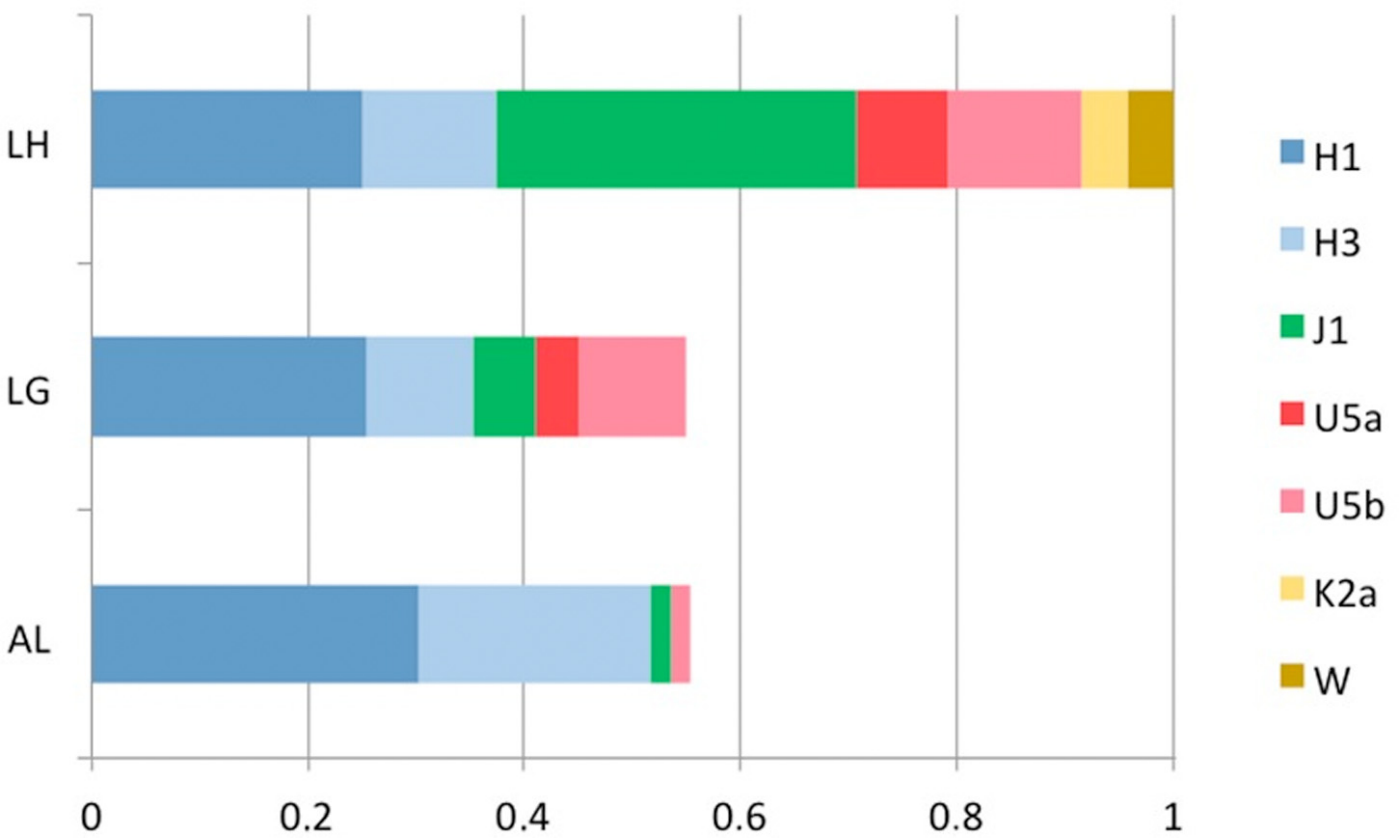
mtDNA haplogroups observed in La Hoya (LH), and their frequencies in the present-day populations studied herein (LG: Laguardia; AL: Alava).

The haplogroup distribution of the extinct and extant populations under study presents certain similarities (Fig 5). Indeed, H1, H3, and J1c2 haplogroups found in La Hoya are also observed in the two present-day groups. Moreover, U5a and U5b are shared between La Hoya and Laguardia. In the case of Alava, U5 is merely represented by one U5b individual (1.8%). It is remarkable that two haplogroups, i.e. K2a and W, described in the ancient population of La Hoya are not found in the two present-day populations here studied.

### Demographic model testing

We used Approximate Bayesian computation method to investigate the genealogical relationship between La Hoya and the present-day Basque populations of Laguardia and Alava. Two competing scenarios were designed in order to test whether La Hoya and Laguardia exhibit genealogical continuity (Scenario 1) or they are genealogically independent (Scenario 2) (S4 Fig). The latter scenario was the best-supported model under a logistic regression (posterior probability >0.77), indicating the ancient and modern populations most likely evolved independently. The type I error from scenario 2 was 27.2% and type II error was 22.9%. This evaluation of scenarios through the ABC approach showed a non-negligible error rate, probably consequence of the limited sample size and polymorphisms of our sample set. In view of these aspects, to expect higher levels of statistical power would be unrealistic, but the analysis still demonstrates a substantial difference in the models’ posterior probabilities supporting scenario 2.

## Discussion

The reconstruction of population history through the study of present-day population data may not be able to infer important information derived from population replacements, genetic drift or minor migration events occurred in certain past periods. For this reason, ancient DNA (aDNA) studies are of great interest as they provide a direct access to prehistoric and historic genetic information. We have carried out the analysis of the mtDNA control region of ancient remains of 41 individuals recovered from the village of La Hoya, one of the most important Iron Age trade centers in the north of the Iberian Peninsula to trace back its connection to current populations living in the area.

The discovery of well-preserved adult human skeletons from the Celtiberian Iron Age is of great historical and biological importance, since few intact adult remains of the Celtiberian culture have been previously recovered. This is consequence of the funeral rites used in that period for adult individuals, where cremation was the common practice. On the other hand, numerous remains from newborns and infants of the Celtiberian period were also found in La Hoya due to the established ritual to bury those young individuals who prematurely died under the eave of their houses [27]. Consequently, it may be expected that individuals from the same family were closely buried.

The major haplogroup found in the archaeological samples from La Hoya was haplogroup H (9 out of 24), particularly subhaplogroups H1 (six individuals) and H3 (three individuals), whose highest frequencies are observed in the Iberian Peninsula and, in particular, in the north of the Iberian Peninsula [21, 28]. In fact, H1 and H3 were also present in the two modern Basque populations analyzed in this study (H1>25% and H3>9%).

Haplogroup J was the second most relevant lineage in La Hoya, particularly subhaplogroup J1 (eight individuals). Previous studies have proposed haplogroup J as a marker for the spread of Neolithic farmers from Near East around 10,000 years ago [29]. Indeed, haplogroup J (J1 and J1c) has been found in Neolithic remains, especially distributed through West Central Europe [30]. However, the coalescence age points out that certain J lineages may have entered Europe prior to the Neolithic period [31], even though, to date no J lineages have been found in European hunter-gatherers. Presently, the greatest frequencies of haplogroup J within the European context occur in populations from the north of Iberia, especially in the Basque Country, whose frequency reaches 14.6% [19]. Nonetheless, this percentage is lower (3–6%) in the two modern populations of Alava. This reduced frequency of haplogroup J in Alava with regard to the rest of Basque regions might be due to a sampling effect or higher influence of recent admixture of migrations from surrounding areas, which may have been eased by the lack of geographical barriers in the south area of the Basque Country.

In La Hoya, haplogroup U5b has been observed in three individuals. In the Iberian Peninsula we have to travel back until the Mesolithic to find the most ancient remains bearing U5b, two individuals U5b2c1 in La Braña-Arintero (León) [32] and one individual U5b1 in Aizpea (Navarre) [33]. Nowadays, subhaplogroup U5b presents a remarkable frequency in the Franco-Cantabrian region which reaches 15% [10]. Among the modern Basque population sample sets studied herein, subhaplogroup U5b was observed in 9.8% of Laguardia and 1.8% of Alava samples.

Finally, haplogroups K and W were found in one individual each in La Hoya sample set. Haplogroup K can also be found in other archaeological sites of the Basque Country [20] and other sites of the Iberian Peninsula [34], while haplogroup W in Iberia has only been observed in very few individuals so far [35]. In modern Basque populations, this pattern is maintained since haplogroup K shows low frequencies, being represented especially by subhaplogroup K1a [8], but subhaplogroup K2a and W have solely been found in two autochthonous Basque individuals [8, 10], respectively. Accordingly, in the case of Laguardia and Alava sample sets, only the subhaplogroup K1a was observed (<4%).

The results of the principal component analysis based on haplogroup composition of historic and prehistoric Iberian populations, placed La Hoya near to other geographically and/or temporary close archaeological sites. Nevertheless, the reduced number of individuals in all ancient sample sets has to be considered cautiously for further relevant conclusions regarding population interrelations.

The study of mtDNA control region of the geographically close populations, Laguardia and Alava, has provided some clues about the possible maternal genetic imprint of La Hoya inhabitants in present-day populations. In terms of mtDNA haplotypes, no exact match was found between the ancient and modern populations. Other mitochondrial aDNA studies have also faced the difficulty of finding identical haplotypes between ancient and modern populations [36]. Phylogenetically close haplotypes within J1c2 lineage were identified among La Hoya, Laguardia, and Alava populations differing in two positions, 16278T and 16366T, which were observed in the current populations. The 16278T variant within J1c lineage has not been observed in ancient remains so far and it appears mainly in modern Basque populations from the Basque Country and Basque Diaspora populations in the American continent (http://empop.online/). The 16366T variant, not found in La Hoya lineages, has been detected in two ancient individuals belonging to J1c2e lineage from the Unetice culture (2,200–1,550 B.C.) and Corded Ware culture (2,500–2,050 B.C.) [37,38]. These evidences lead us to consider different scenarios regarding the J1c2 haplotype (16069T, 16126C, 73G, 185A, 188G, 228A, 263G, 295T) found in La Hoya and its connection to the modern Laguardia population studied: a) the lineage is not represented in the sample analyzed herein; b) the ancient lineage became extinct through generations and, consequently, they are not preserved in the gene pool of contemporary populations; or c) ancient and modern lineages are genealogically independent due to a different origin. Besides, the presence of more substitutions in the modern populations may be explained by the accumulation of mutations through time due to the high mutation rate of the mtDNA control region [13]. Furthermore, genetic drift as well as more recent admixture events occurred in the Alava region needs to be taken into consideration since they might have covered up the maternal genetic imprints of La Hoya in Laguardia population.

Comparative analyses of La Hoya and the present-day populations revealed so far that mitochondrial DNA haplogroups of La Hoya inhabitants are contained in the contemporary European genetic pool, and especially showing a distinctive frequency pattern in the autochthonous populations of the north of the Iberian Peninsula. Despite the archaeological evidences, the apparent genetic similarity of La Hoya and Laguardia, in terms of phylogenetically close lineages and haplogroup composition, is not enough to imply that modern Laguardia population could be the descendant of the once-living in La Hoya. In fact, ABC analysis seems to support the lack of genealogical continuity between these populations at the haplotype level. Nevertheless, the highly similar subhaplogroup composition detected between La Hoya and Laguardia populations do not allow us to reject a maternal genetic continuity in the human groups of the area since at least the Iron Age to present times.

A more comprehensive study based on a larger collection of samples and genetic markers would help to further deepen into more local scale population events, like the founding of Laguardia by the former inhabitants of La Hoya.

## Supporting Information

S1 Fig Map of the archaeological site of La Hoya (Sector I). Location of the infant human remains found is shown by red dots.
(TIF)

S2 Fig Map of the archaeological site of La Hoya (Sector II). Location of the infant human remains found is shown by red dots.
(TIF)

S3 Fig Map of the archaeological site of La Hoya (Sector III). Location of the infant human remains found is shown by red dots.
(TIF)

S4 Fig Graphical representation of the two scenarios examined using approximate Bayesian computation implemented in DIYABC. Scenario 1 corresponds to a genealogical continuity hypothesis between the ancient population of La Hoya and the modern population of Laguardia, and Alava not derived from this ancient population. Scenario 2 assumed that the ancient and modern populations are genealogically independent.
(TIF)

S1 Table Sequence, size (bp) and source of each primer used for PCR amplification and sequencing of the ancient samples of La Hoya. Primers information for coding region positions 7028 and 11467 studied in some modern samples is also shown.
(XLSX)

S2 Table Ancient and modern populations from the literature used to population comparisons. Chronology, population acronym, number of individuals and source reference are shown.
(XLSX)

S3 Table Quantitative PCR (qPCR) results for La Hoya samples. Type of sample (adult or child), DNA concentration (ng/μl) and inhibition level (IPC) for each sample are shown.
(XLSX)

S4 Table mtDNA haplotypes of La Hoya samples. (XLSX)

S5 Table mtDNA haplotypes of Laguardia (N = 51) and Alava province (N = 56). (XLSX)

S6 Table Genetic distances based on F_ST_ values (above the diagonal) with correspondig p values (below the diagonal; 1023 permutations, significance level = 0.05). Genetic diversity (H) is shown.
(XLSX)
