# Supplementary figures and images for: Mitochondrial DNA Reveals the Trace of the Ancient Settlers of a Violently Devastated Late Bronze and Iron Ages Village
Source: PLoS One. 2016 May 13;11(5):e0155342. doi: 10.1371/journal.pone.0155342 (PMC4866787; doi:10.1371/journal.pone.0155342)

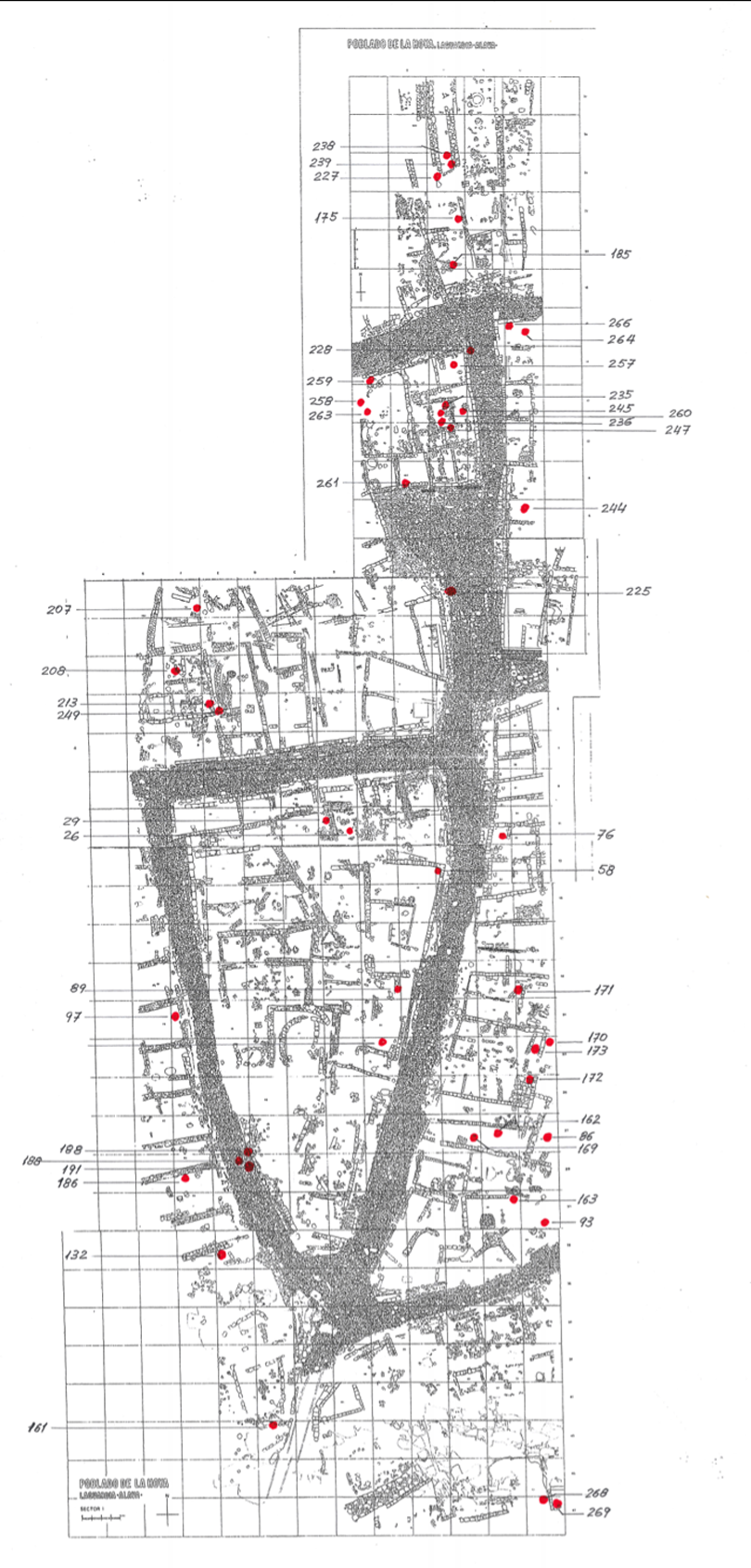

Supplement: S1 Fig — Location of the infant human remains found is shown by red dots. (TIF) [file pone.0155342.s001.tif]

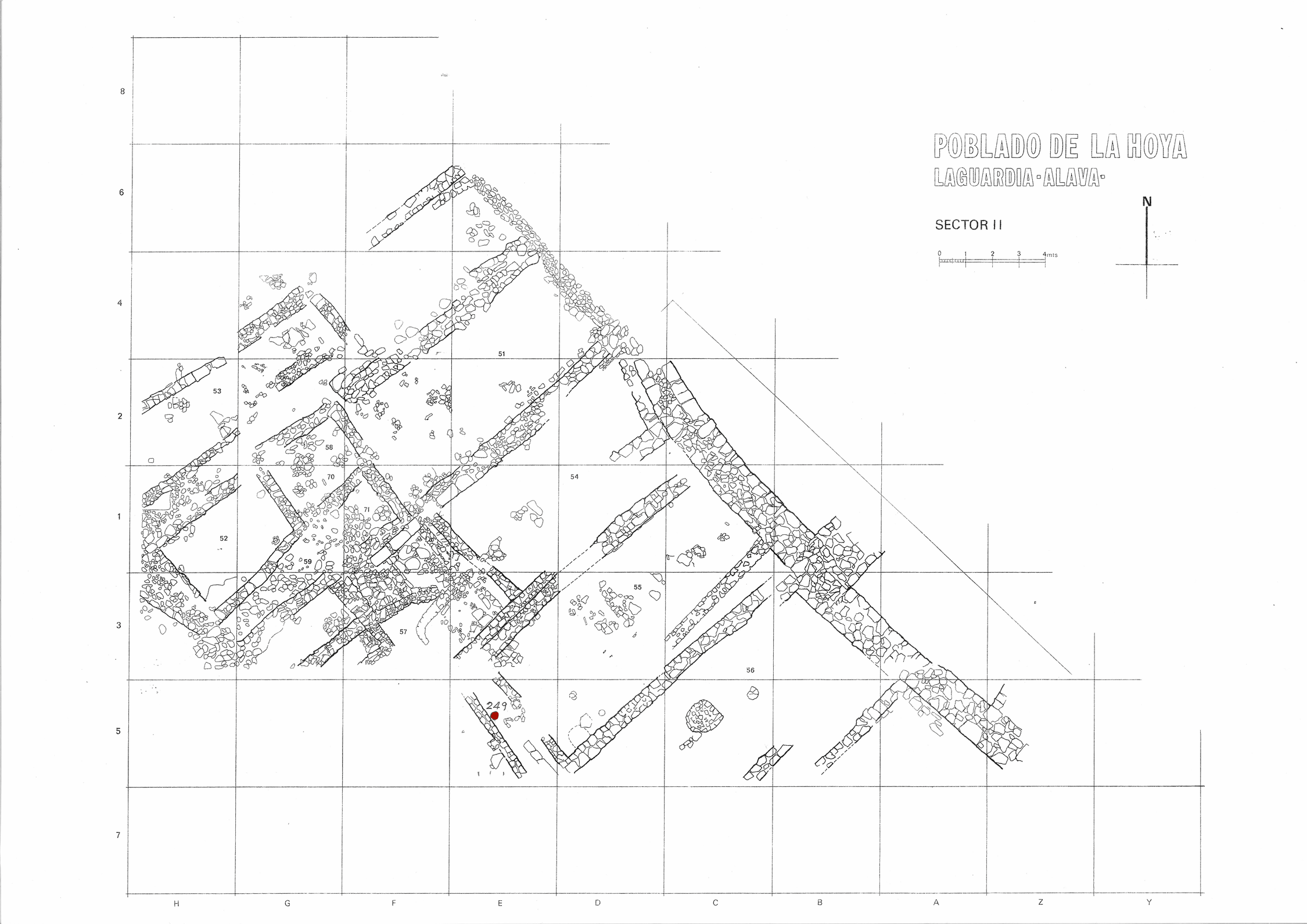

Supplement: S2 Fig — Location of the infant human remains found is shown by red dots. (TIF) [file pone.0155342.s002.tif]

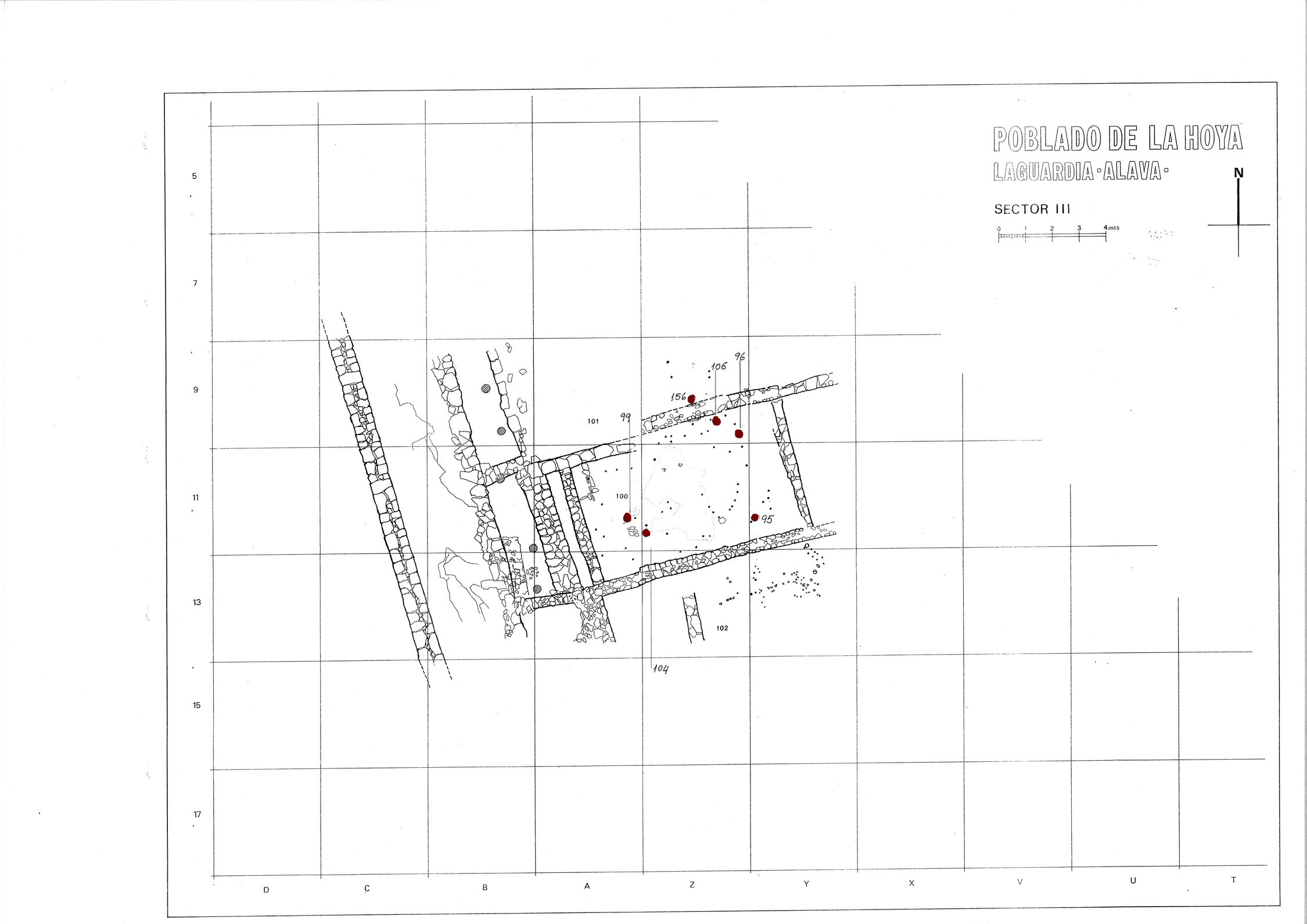

Supplement: S3 Fig — Location of the infant human remains found is shown by red dots. (TIF) [file pone.0155342.s003.tif]

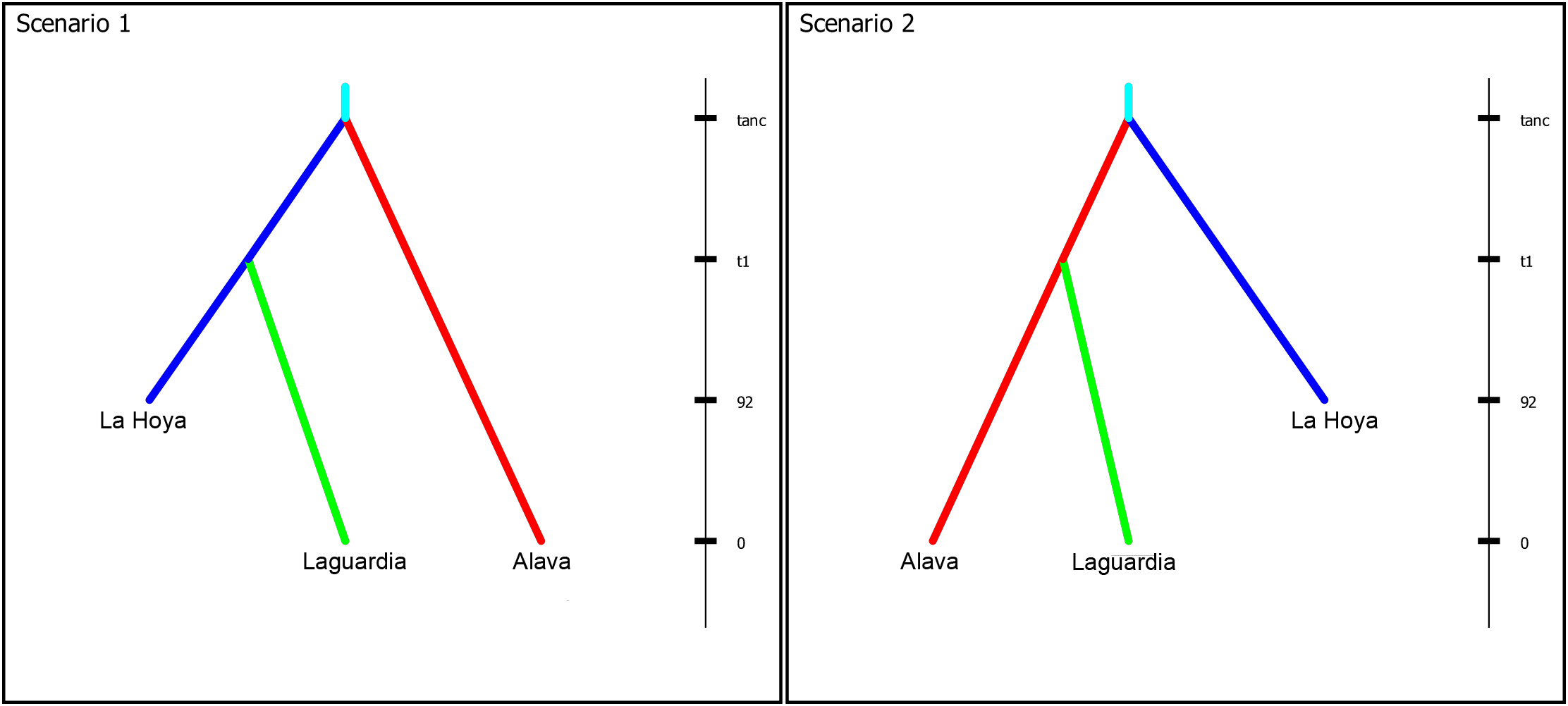

Supplement: S4 Fig — Scenario 1 corresponds to a genealogical continuity hypothesis between the ancient population of La Hoya and the modern population of Laguardia, and Alava not derived from this ancient population. Scenario 2 assumed that the ancient and modern populations are genealogically independent. (TIF) [file pone.0155342.s004.tif]
